# Patterns of attentional biases in children and emotional symptoms during the COVID-19 pandemic: a two-wave longitudinal study

**DOI:** 10.1186/s13034-023-00594-y

**Published:** 2023-05-17

**Authors:** Qiaochu Zhang

**Affiliations:** Department of Social and Behavioural Sciences, College of Humanities and Social Sciences, City University of Hong Kong, Floor 7, AC1Tat Chee Avenue, Kowloon, Hong Kong SAR China

**Keywords:** Latent profiles, Attentional bias, Anxiety symptoms, Depression symptoms, Fear of COVID-19, Children

## Abstract

**Background:**

It is unknown how the patterns of negative and positive attentional biases in children predict fear of COVID-19, anxiety symptoms, and depression symptoms during the COVID-19 pandemic. The study identified profiles of negative and positive attentional biases in children and examined their association with emotional symptoms during the COVID-19 pandemic.

**Method:**

264 children (girls: 53.8% and boys: 46.2%) of 9–10 years born in Hong Kong or mainland China from a primary school in Shenzhen, People’s Republic of China were involved in a two-wave longitudinal study. Children completed the COVID-19 Fear Scale, the Revised Child Anxiety and Depression Scale, and the Attention to Positive and Negative Information Scale to measure fear of COVID-19, anxiety and depression symptoms, and negative and positive attentional biases in classrooms. After six months, they completed the second assessment of fear of COVID-19, anxiety symptoms, and depression symptoms in classrooms. Latent profile analysis was conducted to reveal distinct profiles of attentional biases in children. A series of repeated MANOVA was performed to examine the association of profiles of attentional biases to fear of COVID-19, anxiety symptoms, and depression symptoms across 6 months.

**Results:**

Three profiles of negative and positive attentional biases were revealed in children. Children with a “moderate positive and high negative attentional biases” profile had significantly higher fear of the COVID-19 pandemic, anxiety symptoms, and depression symptoms than children with a “high positive and moderate negative attentional biases” profile. Children with a “low positive and negative attentional biases” profile were not significantly different in fear of COVID-19, anxiety symptoms, and depression symptoms than those with the other two profiles.

**Conclusions:**

Patterns of negative and positive attentional biases were related to emotional symptoms during the COVID-19 pandemic. It might be important to consider children's overall patterns of negative and positive attentional biases to identify children at risk of higher emotional symptoms.

**Supplementary Information:**

The online version contains supplementary material available at 10.1186/s13034-023-00594-y.

## Background

The COVID-19 pandemic might lead to an increase in the rate of emotional symptoms among children [20]. Previous research demonstrated increased fear of COVID-19 and heightened anxiety symptoms and depression symptoms in children with a mean age of 14 years or 10 years during the COVID-19 pandemic [22, 34]. Based on the cognitive models, the tendency to attend to threatening or awarding information is one of the cognitive styles, contributing to emotional symptoms, including fear, anxiety symptoms, and depression symptoms [33]. Although research has demonstrated that low positive attentional bias and high negative attentional bias are risk factors underlying the emotional problems during the COVID-19 pandemic, there are insufficient studies that take a holistic approach and investigate individual differences in their patterns of attending to positive and negative information. Without a holistic perspective, it is unknown if a person who is high in both positive and negative attentional biases develops higher or lower emotional symptoms than a person who is low in both positive and negative attentional biases. With a holistic approach to investigating the overall patterns of attentional biases, the findings would help contribute to the current literature on whether negative attentional bias or positive attentional bias is more important in the development of emotional symptoms. If the profile of high positive and high negative attentional bias was related to low emotional symptoms than was the profile of low positive and low negative attentional biases, positive attentional bias might be more important than negative attentional bias in predicting emotional symptoms. This would provide important implications for the current cognitive models of emotional symptoms, which highlight negative attentional bias but ignore attention to positive information [19].

Longitudinal studies that examine how the profiles of attentional preferences affect emotional symptoms during the COVID-19 pandemic are particularly scarce. Considering the importance of attentional bias in children’s development of internalizing symptoms, more research is needed to investigate how children’s overall patterns of attentional biases might affect the development of fear, anxiety symptoms, and depression symptoms during the COVID-19 pandemic. These findings would add to the literature what were the overall patterns of negative and positive attentional biases in children during the COVID-19 pandemic, and how these patterns of negative and positive attentional biases were associated with emotional symptoms during the COVID-19 pandemic. Such findings might also add to our understanding of whether psychologists should consider both negative and positive attentional biases simultaneously when investigating the development of emotional symptoms in children, especially during the COVID-19 pandemic.

To address these research gaps, the present study aimed to reveal profiles of attentional biases and to examine how these profiles were associated with the development of fear of COVID-19, anxiety symptoms, and depression symptoms in children during the COVID-19 pandemic.

### Attentional bias and internalizing symptoms

Attentional bias refers to the cognitive tendency to shift attention toward negative or positive stimuli. Based on the cognitive models, negative attentional bias is the vulnerability factor for the development of anxiety symptoms and depression symptoms in children [19, 33]. Research recruited 1291 children of 6–18 years with a mean age of 13.5 years, including non-selected youth, high-trait anxiety youth, and treatment-seeking anxiety patients, worldwide and assessed their negative attentional bias through behavioral tasks [1]. The study revealed a significant positive association between negative attentional bias and anxiety symptoms. The association between negative attentional bias measured by the dot-probe task and depression symptoms was also positive in 161 children of 9 to 17 years who were pure depressed, pure anxious, comorbid depressed and anxious, or healthy controls [10]. Another study that measured positive attentional bias in children of 7 to 17 years by the eye-tracking technique found that compared to non-anxious children (N = 49), anxious children (N = 43) had lower attention toward positive information [9]. For depression symptoms, a meta-analysis involving 16 studies found that depressed people over 18 years (366 depressed patients, and 563 health controls) had impaired positive attentional bias [28]. A longitudinal study showed that low positive attentional bias measured by spatial orienting tasks might cause increased depression symptoms for never-depressed adolescents (N = 531,81 developed depression during follow-up of 16 years [29]. The above studies measured attentional bias by behavioral tasks, such as the dot-probe task or the spatial orienting task. Although behavioral tasks are objective measures of attentional bias, these have limitations in their poor psychometric properties which include poor test–retest reliability or convergent validity [3, 30]. Therefore, other methods to assess attentional bias might be needed to increase our understanding of attentional bias. For negative attentional bias, a longitudinal study revealed that high negative attentional bias measured by the self-report inventory predicted the developmental trajectories of high anxiety symptoms in 72 Chinese children from grade 9 to 11 in high schools over three years [11]. The self-report inventory of attentional bias such as the Attention to Positive and Negative Information Scale had good reliabilities and validities [23]. However, the self-report measure of attentional bias was often criticized for its susceptibility to memory bias [25].

Attentional training that targeted negative attentional bias has shown efficacy in modifying self-esteem as an indicator of depression symptoms in 108 participants over 17 years old with mild or minimal depression symptoms (63 females, 45 males) [17]. Also, a study tested the efficacy of attentional bias modification in reducing negative attentional bias and social anxiety in 32 healthy adolescents aged 13–16 years from secondary schools in the Netherlands. Compared to adolescents who completed the placebo training, those who conducted attentional bias modification had reduced negative attentional bias and social anxiety symptoms [8]. This evidence further supported that high negative attentional bias might affect high anxiety symptoms and depression symptoms.

During the COVID-19 pandemic, children around the age of 14.23 years (N = 195) recruited from the Netherlands might experience strong fear of COVID-19 infection which was the result of communication of threatening COVID-19-related information with parents [22]. A meta-analysis has suggested that higher attention toward threats was related to higher fear-related symptoms in adult participants, including clinical, community, or student samples, in 40 studies [7]. Thus, attentional bias toward threats might also be related to higher fear of COVID-19. Moreover, a longitudinal study on 132 Italian adults who experienced lockdown due to the COVID-19 pandemic in Italy found that higher attention toward COVID-related threatening stimuli was associated with higher health anxiety symptoms [4]. Higher attention to COVID-19-related stimuli was associated with higher COVID-19 anxiety syndrome in 150 females and 148 males who were UK residents above 18 years [2]. Therefore, research on attentional bias during the COVID-19 pandemic might provide important implications for identifying children who are more likely to show stronger fear of COVID-19, anxiety symptoms, and depression symptoms during the COVID-19 pandemic.

### Profiles of negative and positive attentional biases

Profiles of negative and positive attentional biases depicted overall patterns of attentional preferences to negative and positive stimuli. Although negative attentional bias might be the opposite of positive attentional bias in terms of the affective valence of preferred stimuli, people might have low negative attentional bias and low positive attentional bias simultaneously. The presence of high negative and high positive attentional biases might predict lower emotional symptoms than those with high negative and low positive attentional biases. For example, a study with an unselected community sample of 270 adults (the mean age of 22 years) from the undergraduate student population and community forum has shown that attentional bias toward threats was linked to high self-report anxiety symptoms only in individuals with a tendency to attend away from positive information [32]. Latent profile analysis is a person-centered statistical approach that is commonly used to reveal heterogeneous groups of people with common patterns of external or internal behavior [24]. A cross-sectional study assessed attentional biases by self-report inventories in 667 inpatients of 60–90 years from a hospital in China and revealed four distinct profiles of positive and negative attentional biases with latent profile analysis [13]. The four profiles consisted of “no positive and negative bias” which included 9.3% of participants, “minor positive bias & no negative bias” (48.0%), “major positive bias & minor negative bias” (25.6%), and “major positive bias & no negative bias” (17.1%). The “no positive and negative bias” and the “minor positive bias & no negative bias” groups had higher depression symptoms than the “major positive bias & no negative bias” group, suggesting that high positive attentional bias was a protective factor buffering depression symptoms. However, it is unclear if the results can be replicated in children during the COVID-19 pandemic. Moreover, the study is cross-sectional, which has limited ability to imply causality. Thus, more longitudinal research on children’s profiles of attentional biases is needed to understand how profiles of attentional bias affect fear of COVID-19, depression symptoms, and anxiety symptoms during the COVID-19 pandemic. Profiles of negative and positive attentional biases would provide clinical implications for identifying children who are at high risk of developing strong fear of COVID-19, anxiety symptoms, and depression symptoms based on their profiles of attentional biases during the COVID-19 pandemic. Also, the profiles would imply that clinicians should address profiles of negative and positive attentional biases when preventing children from development of emotional symptoms during the COVID-19 pandemic.

### The current study

The current research recruited a sample of children from a primary school. The study aimed to 1) reveal distinct profiles of negative and positive attentional biases in children during the COVID-19 pandemic. Also, the study aimed to 2) reveal the relationship of profiles of attentional biases to fear of COVID-19, anxiety symptoms, and depression symptoms during the COVID-19 pandemic. The majority of existing studies have only investigated either negative attentional bias or positive attentional bias separately. No previous studies have used the latent profile analysis to investigate the effect of the overall patterns of attentional biases on children’s emotional symptoms during the COVID-19 pandemic. This study would be the first study that took a holistic approach to reveal profiles of attentional biases in children. Also, it would provide the first evidence from a longitudinal study for how profiles of attentional biases would affect fear of COVID-19, anxiety symptoms, and depression symptoms during the COVID-19 pandemic.

Based on the previous evidence, distinct profiles of attentional biases would be revealed which might include profiles of “low negative and positive attentional bias”, “low negative and high positive attentional bias”, “high negative and low positive attentional bias” and “high negative and positive attentional bias”. Compared to the “high negative and positive attentional bias” profile, the “low negative attentional bias and high positive attentional bias” profile would predict low fear, anxiety symptoms, and depression symptoms during the COVID-19 pandemic. While compared to the “low negative and positive attentional bias” profile, the “high negative attentional bias and low positive attentional bias” profile would be related to more fear, anxiety symptoms, and depression symptoms in children during the COVID-19 pandemic.

## Method

### Participants

The study initially recruited a sample of middle-class 322 children from a primary school in Shenzhen, PRC. Children had similar social economic status and education levels. They were from middle-class families and had the same grade at the same school.

Convenient sampling was used to recruit participants. First, public schools where children were normal Chinese students from middle-class families were selected. This population was chosen because the middle class is rapidly growing, and takes a significantly large proportion of the population in China [27]. Then, invitation letters to collaborate were sent to the selected schools by email. The school would reply to us whether they would allow us to recruit participants in the schools. The study was conducted in the school which permitted the recruitment of students. Teachers played the role of coordinators. They helped researchers coordinate with students who were interested in participating in the study. Teachers announced the details of the study and disseminated information sheets, consent, and assent forms to students in the classrooms. Also, teachers would help researchers exclude students who didn’t fulfill the criteria to participate in the study.

Regarding gender distribution, 53.8% (N = 171) of participants were girls, and 46.2% (N = 147) were boys; three participants did not indicate their gender. The average age of the sample was 9.54 years, ranging from 9 to 10 years (SD = 0.505 years). Exclusion criteria were (1) not able to fluently speak and understand simplified Chinese, (2) having a diagnosis of psychological disorders or developmental disorders, and (3) birthplace outside of mainland China. 50 children were excluded from the study because they failed to meet the inclusion criteria. 8 children who failed to complete one of the self-report assessments were excluded from the research. Altogether, 58 students were excluded from the study. Consequently, data from 264 children (N _boys_ = 124 and N _girls_ = 138) were input into analyses.

### Procedure

A preliminary study was first conducted to validate the Attention to Positive and Negative Information Scale in 122 children of 11–13 years from a primary school (mean age of 11.90 years). First, researchers assigned the informed consent and assent forms to students. After collecting the informed consent and assent forms from students, children completed the Attention to Positive and Negative Information Scale for approximately 30 min in the classrooms. Researchers answered questions regarding the scale from students in the classrooms.

The present study was conducted from November 16th to November 30th, 2020, when the COVID-19 pandemic was ongoing. The prevention measures, including social distancing, were implemented during the period. Teachers in the primary school helped researchers disseminate informed consent forms and assent forms to parents and students. The signed informed consent and assent forms were collected one day before the date of data collection in the classrooms. Only children who signed the assent forms and obtained the signed informed consent forms from their parents participated in the study. On the data collection date, research assistants assigned a set of questionnaires to students during classes. The participants were informed that they could ask questions and withdraw from the study at any time without any consequence. The assessment of attentional biases was conducted concurrently with the first assessment of emotional symptoms at time one (T1) and before the second assessment of emotional symptoms at time two (T2). At time one, students completed a set of questionnaires for children to assess their negative and positive attentional biases, fear of COVID-19, anxiety symptoms, and depression symptoms for approximately 45 min in the classrooms. After approximately six months, students completed the questionnaire to assess their fear of COVID-19, anxiety symptoms, and depression symptoms at time two in the classrooms for approximately 45 min. In each assessment, a teacher and researcher were present to answer students’ questions and ensure the safety of students.

## Measurement

### Negative and positive attentional biases

The eight-item Chinese version of the Attention to Positive and Negative Information Scale (CAPNIS) was administered to children [5, 23]. The scale had good structural validity. Refer to the Additional File 1 for the exploratory factor and confirmatory factor analysis of the scale. The internal consistency in the current study was satisfactory (API = 0.69; ANI = 0.76). Also, the scale showed good current validity as ANI and API had significant associations with anxiety symptoms and depression symptoms at T1 and T2 (Refer to Table 1). The scale consisted of an Attention to Negative Information subscale (ANI; four items) and an Attention to Positive Information (API; four items) subscale, rated on a five-point Likert scale. A negative attentional bias score was calculated by summing up scores from the ANI subscale. A positive attentional bias score was obtained by adding scores from the API subscale. Higher scores indicated higher attentional biases.

**Table 1:** Mean (Standard Deviation) and correlations of psychological variables (n = 262)

	Girls		Boys		Correlations							
	Means	SD	Means	SD	*r*	*r*	*r*	*r*	*r*	*r*	*r*	*r*
					T1 ANI	T1 API	T1 anxiety symptoms	T1 depression symptoms	T1 Fear of COVID-19	T2 anxiety symptoms	T2 depression symptoms	T2 Fear of COVID-19
T1 ANI	11.51	4.05	10.80	3.75	–							
T1 API	14.86	3.09	14.88	3.46	− 0.099	–						
T1 anxiety symptoms	29.55	23.23	21.46	17.86	0.679**	− 0.178**	–					
T1 depression symptoms	5.75	5.74	4.81	4.95	0.619**	− 0.210**	0.868**	—				
T1 Fear of COVID-19	14.69	11.39	11.79	10.52	0.443**	− 0.160*	0.610**	0.509**	–			
T2 anxiety symptoms	29.11	21.67	21.80	18.55	0.502**	− 0.180**	0.721**	0.655**	0.572**	–		
T2 depression symptoms	6.51	5.87	5.05	5.00	0.451**	− 0.224**	0.595**	0.651**	0.473**	0.852**	—	
T2 Fear of COVID-19	28.29	11.29	24.30	10.58	0.408**	− 0.071	0.476**	0.422**	0.635**	0.628**	0.579**	–

^*^ *p* < .05 ** *p* < .01; API: Attention to Positive Information; ANI: Attention to Negative Information; T1: Time one; T2: Time two; SD: Standard Deviation

### Emotional symptoms

#### Anxiety symptoms and depression symptoms

The Chinese version of the Revised Child Anxiety and Depression Scale [6] was used to measure children’s anxiety symptoms and depression symptoms. The Chinese version could be downloaded from the website (http://www.childfirst.ucla.edu/Resources.html). RCADS is a wildly used scale for childhood anxiety symptoms and depression symptoms. The 47 items of the scale can be grouped into the Anxiety Disorders and the Major Depression sub-scales, rated on a Likert scale from 0 to 3 (0 = “never”; 1 = “sometimes”; 2 = “often”; and 3 = “always”). The scale of Anxiety Disorders has 37 items (e.g., “afraid of looking foolish in front of people”), and the scale of Major Depressive includes 10 items (MDD; “feels nothing is much fun anymore”). An anxiety symptoms score and a depression symptoms score were calculated by summing up scores of the relevant items on the subscales of Anxiety Disorder and Major Depression, respectively. The scales showed excellent reliability with the current data (Time one: Anxiety Disorder Cronbach’s Alpha = . 95; Major Depression Cronbach’s Alpha = 0.88; Time two: Anxiety Disorder Cronbach’s Alpha = . 96; Major Depression Cronbach’s Alpha = 0.88).

#### Fear of COVID-19

Fear of COVID-19 was measured by the COVID-19 Fear Scale (CFS). CFS was adapted from the Chinese version of the 18-item SARS Fear Scale (SFS; Ho, Kwong-Lo, Mak, & Wong, 2005). When adapting the CFS scale, three items were deleted from the original scale, because these items were only related to healthcare workers (“Feel distressed because of the upsurge in workload”, “Worry if my family or friends will keep a distance from me due to my job duties”, and “Worry if I will be assigned to SARS wards”). Also, instead of SARS, participants were asked about their response to the COVID-19 pandemic in the COVID-19 Fear Scale.

Participants were asked to rate on a 4-point Likert scale how much the statement is true in the face of the COVID-19 pandemic. 0 indicates "definitely false" and 3 indicates "definitely true." The sample item includes "Fear that I will be infected." Three items were excluded from CFS ("Worry if I will be assigned to COVID-19 wards", "Feel distressed because of the upsurge in workload," and "Worry if my family or friends will keep a distance from me due to my job duties") because of their inapplicability to students. A CFS score was obtained by summing up scores from each item. The higher the CFS scores, the higher the fear of COVID-19. The scale demonstrated good internal consistency in the current study (T1 Cronbach's α = 0.94; T2 Cronbach's α = 0.95).

### Data analysis

Exploratory factor analysis was first performed to examine the structural validity of the Attention to Positive and Negative Information Scale with data from the preliminary study (Refer to Additional file 1 Information for details). Then, confirmatory factor analysis (CFA) was conducted to confirm the structural validity of the eight-item Chinese version of the APNIS (CAPNIS) with the sample of the current study (N = 264). (Refer to Additional file 1 Information for details).

Descriptive statistics were presented. Two-tailed Pearson’s partial correlation was investigated using SPSS version 22 to understand the association among psychological variables. Independent t-tests examined the gender differences in the psychological variables. Latent profile analyses were subsequently conducted to reveal profiles of negative and positive attentional biases in children with MPlus 7.4. With maximum likelihood estimation with robust standard error (MLR), heterogeneous profiles of attentional biases were revealed. The number of groups for the model was increased until the model was not significantly better than the model with one fewer group. Smaller values of the Bayesian Information Criterion (BIC) and Akaike information criteria (AIC), as well as the higher value of entropy, suggested a better model fit [14]. The significant *p* values of the Vuong–Lo–Mendell–Rubin likelihood ratio test (VLMR), the adjusted Lo–Mendell–Rubin likelihood ratio test (Adj. LMR), as well as the Bootstrapped Likelihood ratio test (BLRT), showed that a model with k number of groups was significantly better than a model with k-1 groups [14]. Next, Chi-Square analyses assessed if groups with distinct profiles of attentional biases differed in gender, age, and birthplace. Three repeated multivariate analyses of variance (MANOVA) were conducted to examine the effect of time, profiles of negative and positive attentional biases, as well as the interaction between profiles and time on anxiety symptoms and depression symptoms over 6 months. Bonferroni post hoc tests were further conducted to compare the level of fear of COVID-19, anxiety symptoms, and depression symptoms among the latent profiles. The Bonferroni test was the most frequently used post hoc test and was recommended for pairwise comparisons in parameter situations by researchers [12, 18]. The datasets generated during and/or analyzed during the current study are available from the corresponding author upon reasonable request.

## Results

### Descriptive statistics

An independent t-test was performed with 262 participants to examine the difference between girls and boys in positive and negative attentional biases, fear of COVID-19, depression symptoms, and anxiety symptoms assessed at time one (T1) and time two (T2). Results revealed that girls and boys didn’t significantly differ in negative attentional bias,* t* (260) = 1.48, *p* = 0.14, positive attentional bias, *t* (260) = − 0.04, *p* = 0.97, and depression symptoms at T1, *t* (260) = 1.41, *p* = 0.16. Girls showed significantly higher anxiety symptoms at T1, *t* (260) = 3.13, *p* < 0.005, higher anxiety symptoms at T2, *t* (260) = 2.92, *p* < 0.005, and higher depression symptoms at T2, *t* (260) = 2.17, *p* < 0.05, than did boys. Also, girls were significantly higher in fear of COVID-19 at T1, *t* (257) = 2.12, *p* < 0.05, than boys. Girls were significantly higher in fear of COVID-19 at T2, *t* (256) = 2.92, *p* < 0.005. Refer to Table 1 for the means and standard deviations of each variable by gender.

The Pearson’s partial correlation, controlling for gender revealed that age was not significantly associated with positive attentional bias, *r* = − 0.01, *p* = 0.82, negative attentional bias, *r* = 0.04, *p* = 0.57, anxiety symptoms at T1, *r* = − 0.04, *p* = 0.52, depression symptoms at T1, *r* = − 0.03, *p* = 0.61, anxiety symptoms at T2, *r* = − 0.03, *p* = 0.60, and depression symptoms at T2, *r* = − 0.02, *p* = 0.76.

Higher anxiety symptoms at T1 and T2 were significantly related to higher depression symptoms at T1 and T2. They were also significantly associated with higher negative and lower positive attentional biases. Depression symptoms at T1 and T2 were positively and significantly associated with negative attentional bias; depression symptoms at T1 and T2 were negatively and significantly related to positive attentional bias. Higher fear of COVID-19 at T1 and T2 was associated with significantly higher anxiety symptoms and depression symptoms at T1 and T2. Also, higher fear of COVID-19 at T1 and T2 was significantly related to higher negative attentional bias. Higher fear of COVID-19 at T1 was significantly associated with lower positive attentional bias. Higher fear of COVID-19 at T2 was not significantly related to lower positive attentional bias. Higher anxiety symptoms at T1 were significantly related to higher anxiety symptoms at T2; higher depression symptoms at T1 were significantly related to higher depression symptoms at T2. Fear of COVID-19 at T1 was significantly related to higher fear of COVID-19 at T2. Negative attentional bias was not significantly associated with positive attentional bias. Refer to Table 1 for correlation coefficients.

### Profiles of negative and positive attentional biases at T1

The fit indices for models with one to five groups were examined with a latent profile analysis (refer to S.Table 1 in Additional file 1 information for fit indices). The three-class model had the smallest BIC and AIC. The VLMR, Adj.LMR and BLRT tests were all significant for the three-class model. All *p* values of VLMR, Adj. LMR and BLRT were insignificant for the four-class model, which suggested that the four-class model was not significantly better than the three-group model. Thus, the three-class model showed the best model fit.

**Fig. 1:**
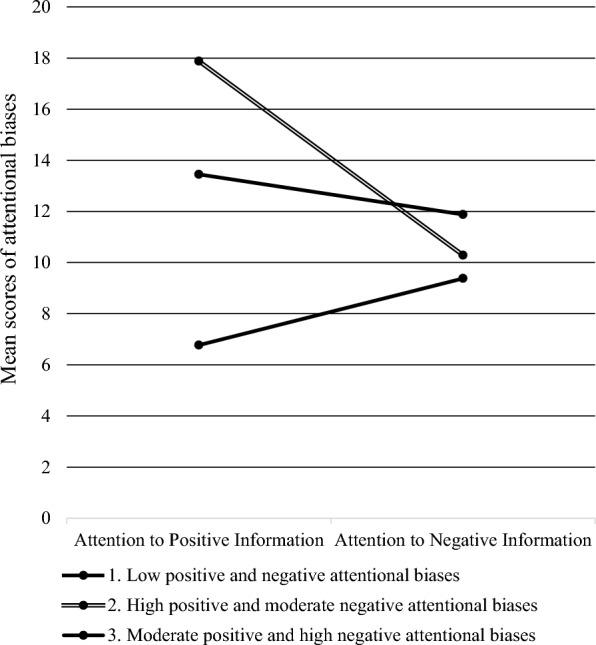
Mean scores of positive attentional bias and negative attentional bias in the three profiles of attentional biases

Class one had the lowest level of negative and positive attentional biases (Mean _negative attentional bias_ = 8.60; Mean _positive attentional bias_ = 6.40), and was named as “low positive and negative attentional biases” profile (3.8%; Mean _age_ = 9.50). Class two had the highest positive attentional bias and moderate negative attentional bias (Mean _negative attentional bias_ = 10.06; Mean _positive attentional bias_ = 18.18), which was accordingly defined as the “high positive and moderate negative attentional biases” profile (36.7%; Mean _age_ = 9.56). Class three had a moderate positive attentional bias and high negative attentional bias (Mean _negative attentional bias_ = 12.01; Mean _positive attentional bias_ = 13.36), which was named the “moderate positive and high negative attentional biases” profile (59.5%; Mean _age_ = 9.57). Figure 1 shows the means of negative and positive attentional biases of the three profiles.

The three latent profiles were not significantly different in terms of gender, *χ*^*2*^ (2) = 0.23, *p* = 0.89, birthplace, *χ*^*2*^ (2) = 1.14, *p* = 0.57, and age groups (9 & 10 years), *χ*^*2*^ (2) = 1.24, *p* = 0.54. Refer to Table 2 for information on gender, birthplace, and age groups in the three profiles.

**Table 2:** Number and percentages of gender, age, and birth place based on the three profiles of attentional biases

Profiles	Low negative and positive attentional bias N = 10 (3.8%)	High positive and moderate negative attentional bias N = 97 (36.7%)	3. Moderate positive and high negative attentional bias N = 157 (59.5%)
Gender			
Girls	5 (50%)	46 (47.9%)	87 (55.8%)
Boys	5 (50%)	50 (52.1%)	69 (44.2%)
Age			
9	5 (50%)	42 (44.2%)	67 (42.9%)
10	5 (50%)	53 (55.8%)	89 (57.1%)
Birth place			
Mainland	10 (100%)	87 (89.7%)	146 (93%)
Hong Kong	0 (0%)	10 (10.3%)	11 (7%)

### The relationship of profiles of attentional biases to emotional symptoms

#### Anxiety symptoms

A repeated MANOVA was performed to examine the between-subject effect of profiles and the within-subject effect of time, as well as the interaction effect between profiles and time on anxiety symptoms. Results suggested the within-subject effect of time was insignificant. The interaction between time and profiles was also insignificant. The between-subject effect of profiles on anxiety symptoms was significant, *F* (2,261) = 5.66, *p* < 0.001. The effect size was small, partial η^2^ = 0.042. Power for the effect of profiles on anxiety symptoms was as high as 0.86.The Bonferroni post hoc test showed that children with a “moderate positive and high negative attentional biases” profile had significantly higher anxiety symptoms (Mean _anxiety_ = 28.71; Standard Deviation = 1.52) than children with a “high positive and moderate negative attentional biases” profile (Mean _anxiety_ = 20.54; Standard Deviation = 1.93). Children with a “low positive and negative attentional biases” profile (Mean _anxiety_ = 28.85; Standard Deviation = 6.02) were not significantly different in anxiety symptoms level from children with the other two profiles (refer to Table 3).

**Table 3:** Changes in anxiety symptoms and depression symptoms based on the three profiles of attentional biases

	1. Low negative and positive attentional bias		2. High positive and moderate negative attentional bias		3. Moderate positive and high negative attentional bias		Difference Between profiles	Time effect	Profile * Time
	Means (SD)		Means (SD)		Means (SD)				
	T1	T2	T1	T2	T1	T2	*F*	*F*	*F*
Anxiety symptoms	25.20 (19.33)	32.50 (16.51)	19.95 (19.34)	21.12 (20.94)	29.37 (21.63)	28.06 (20.10)	5.66** 2 < 3	1.89	2.01
Depression symptoms	7.10 (5.93)	8.60 (4.62)	3.93 (4.90)	4.54 (5.19)	6.08 (5.48)	6.45 (5.59)	6.43** 2 < 3	2.49	.34
Fear of COVID-19	13.80 (12.32)	20.60 (9.63)	10.77 (10.14)	24.63 (10.99)	14.76 (11.31)	27.74 (10.99)	4.10*	112.55**	2.64

^*^ *p* < .05 ** *p* < .01; SD: Standard Deviation; T1: Time one; T2: Time two; Profile*time: Interaction between profile and time

#### Depression symptoms

A repeated MANOVA for depression symptoms revealed an insignificant with-in-subject effect of time and an interaction effect of time and profiles. The between-subject effect of profiles was significant,* F* (2,261) = 6.43, *p* < 0.01. The effect size was small with partial η^2^ = 0.047. The between-subject effect had high calculated power of 0.90. Bonferroni post hoc test showed that children with a “moderate positive and high negative attentional biases” profile (Mean _depression_ = 6.26; Standard Deviation = 1.53) had significantly higher depression symptoms than children with a “high positive and moderate negative attentional biases” profile (Mean _depression_ = 4.23; Standard Deviation = 0.49). Children with a “low positive and negative attentional biases” profile (Mean _depression_ = 7.85; Standard Deviation = 0.39) were not significantly different in depression symptoms from children with the other two profiles (refer to Table 3).

#### Fear of COVID-19

With the fear of COVID-19 as the dependent variable and patterns of attentional biases as the independent variable, a repeated MANOVA demonstrated a significant with-in-subject effect of time; fear of COVID-19 significantly increased from T1 to T2. The interaction effect of time and profiles was insignificant. The between-subject effect of profiles was significant,* F* (2,254) = 6.43, *p* < 0.05. The effect size was small, partial η^2^ = 0.031. The calculated power for the effect of profiles on fear of COVID-19 was 0.72. The Bonferroni post hoc test showed that children with a “moderate positive and high negative attentional biases” profile (Mean _fear of COVID-19_ = 21.25; Standard Deviation = 0.80) demonstrated significantly higher fear of COVID-19 than children with a “high positive and moderate negative attentional biases” profile (Mean _fear of COVID-19_ = 17.70; Standard Deviation = 1.02). Children with a “low positive and negative attentional biases” (Mean _fear of COVID-19_ = 17.20; Standard Deviation = 3.13) profile didn’t significantly differ in fear of COVID-19 from children with “moderate positive and high negative attentional biases” and “high positive and moderate negative attentional biases” (refer to Table 3).

## Discussion

The study revealed three profiles of attentional biases that depicted the overall patterns of attention to negative and positive information in children’s daily life: “low positive and negative attentional biases” profile (4.3%), “high positive and moderate negative attentional biases” profile (38.5%), and “moderate positive and high negative attentional biases” profile (57.2%). Profiles of attentional biases showed a significant relationship with emotional symptoms over six months during the COVID-19 pandemic; the “moderate positive and high negative attentional biases” profile was related to significantly higher anxiety symptoms and depression symptoms than “high positive and moderate negative attentional biases” profile. The “low positive and negative attentional biases” profile was not significantly different from the other two profiles in emotional symptoms. Consistent with the findings, previous studies which measured negative attentional bias by behavioral tasks also found a positive association between negative attentional bias and emotional symptoms. [10, 16]. The present finding that the “moderate positive and high negative attentional biases” profile predicted higher anxiety and depression in children than the “moderate negative and high positive attentional biases” profile was consistent with the previous results that low positive attentional bias was associated with high anxiety and depression symptoms [9, 28]. A previous longitudinal study showed that high negative attentional bias predicted the trajectory of high anxiety in children [11]. Interventions that targeted negative attentional bias also suggested that reduced negative attentional bias was associated with low anxiety and depression (e.g., [15]. These were congruent with the findings from the longitudinal studies which further suggested that the “moderate positive attentional bias and high negative attentional bias” profile predicted higher anxiety and depression symptoms than the “high positive attentional bias and moderate negative attentional bias” profile.

Moreover, during the COVID-19 pandemic, negative attentional bias was related to higher health anxiety and higher COVID-19-related anxiety symptoms [2, 4]. This was also reflected in the association between profiles of attentional biases and emotional symptoms during the COVID-19 pandemic. The findings supported that the “moderate positive attentional bias and high negative attentional bias” profile predicted higher fear of the COVID-19 pandemic than the “high positive attentional bias and moderate negative attentional bias” profile. These findings replicated results from the meta-analysis that attentional bias towards threats was associated with higher fear of COVID-19 [7].

These were the first results identifying children’s patterns of attentional biases during the COVID-19 pandemic and their relationship with emotional symptoms. With latent profile analysis and the longitudinal design, the results added to the literature that it was important to consider negative and positive attentional biases simultaneously and to use profiles of attentional biases to predict the development of emotional symptoms during the COVID-19 pandemic. Evidence might provide significant implications for the cognitive model of emotional symptoms, suggesting that negative attentional bias was as important as positive attentional bias, and thus the overall patterns of negative and positive attentional bias might be more important than negative attentional bias alone.

### The patterns of positive and negative attentional biases

The current study revealed three profiles of positive and negative attentional biases in children during the COVID-19 pandemic. Over half of the children showed a “moderate positive and high negative attentional biases” profile (59.5%), and only a smaller number of children had a “low positive and negative attentional biases” profile (3.8%) or “high negative attentional bias and moderate positive attentional bias” profile (36.7%). Consistent with previous research, most children’s attentional bias towards negative stimuli tended to be moderate or high during the COVID-19 pandemic (e.g., [4]. Previous studies have shown that the COVID-19 pandemic harmed children’s mental health and consequently lead to a higher risk of psychopathology in children [26]. Negative attentional bias as a vulnerability factor for anxiety and depression symptoms might be higher because of the stressful situation in the COVID-19 pandemic. This was supported by our findings that as high as 59.5% of children had a “moderate positive attentional bias and high negative attentional bias” profile which accounted for a higher proportion of the sample than the other two profiles. Additionally, the results also added that the majority of children had moderate or high attention to positive stimuli during the COVID-19 pandemic.

These results were inconsistent with the findings from a previous study by Ji et al. [13], which revealed four profiles of attentional biases in adults by the APNIS: “no positive and negative bias” group (9.3%), “minor positive bias & no negative bias” group (48.0%), “major positive bias & minor negative bias” (25.6%), and “major positive bias & no negative bias” (17.1%). While both studies provided evidence that the fewest number of individuals showed zero to low negative and positive attentional biases in adults and children. This evidence supported that the majority of children had moderate to high levels of attentional biases during the COVID-19 pandemic. It should also be noted that for both the previous study by Ji et al. [13] on adults and the current study on children, the profile of high negative and positive attentional biases was not revealed, suggesting that these children hardly show strong attentional bias towards both negative and positive stimuli, even during the COVID-19 pandemic.

### The relationship of profiles of attentional biases to fear of COVID-19, anxiety symptoms, and depression symptoms

Previous research revealed that the “no positive and negative bias” group and “minor positive bias & no negative bias” group were related to higher depression symptoms than the “major positive bias & no negative bias” group [13]. This was not replicated in the current study with children. The “low positive and negative attentional biases” profile was not significantly related to higher depressive symptoms and anxiety symptoms than the other two profiles.

According to previous studies, negative attentional bias is associated with higher fear of COVID-19, anxiety symptoms, and depression symptoms, while positive attentional bias is related to lower fear of COVID-19, anxiety symptoms, and depression symptoms [11, 29]. The cognitive model suggested that negative attentional bias was the vulnerability factor for the development of anxiety symptoms. Consistent with the cognitive theories, the study revealed that children with a “moderate positive and high negative attentional biases” profile had higher anxiety symptoms and depression symptoms than those with “high positive and moderate negative attentional biases”. This suggested that high negative attentional bias predicted high anxiety and depression symptoms in children during the COVID-19 pandemic.

Interestingly, children with a “low positive attentional bias and negative attentional bias” profile didn’t significantly differ in fear of COVID-19, anxiety symptoms, and depression symptoms levels compared to the other two groups. This might suggest that lower negative attentional bias was not significantly related to lower fear of COVID-19, anxiety symptoms, and depression symptoms when positive attentional bias was low, which was consistent with previous results from Wei, Roodenrys, & Miller [32]. Also, lower positive attentional bias might not associate with higher emotional symptoms when negative attentional bias was low. This contributed to our understanding that positive attentional bias might be as important as negative attentional bias when predicting the development of emotional symptoms during the COVID-19 pandemic. Thus, the findings added to the existing literature by highlighting the importance of investigating the general patterns of both positive and negative attentional biases simultaneously during the COVID-19 pandemic.

### Implications

The study revealed three patterns of attentional biases in children and assessed their association with emotional symptoms during the COVID-19 pandemic. The results provided important implications for the cognitive model of emotional disorders. A large proportion of children had the “moderate positive and high negative attentional biases” and the “high positive and moderate negative attentional biases” profiles. These children didn’t significantly differ in the development of emotional symptoms from the minority of children who had “low positive and negative attentional biases” during the COVID-19 pandemic. Thus, the three profiles identified in the study had important clinical implications that profiles of negative and positive attentional biases might be significant predictors of the development of anxiety symptoms and depression symptoms in children; negative attentional bias might be as important as positive attentional bias in predicting emotional symptoms including fear of COVID-19, anxiety symptoms, and depression symptoms during the COVID-19 pandemic. Having high negative attentional bias or low positive attentional bias was not necessarily associated with high emotional symptoms; consequently, it is important to consider the overall patterns of positive and negative attentional biases. Indeed, inconsistent evidence on the relationship between negative attentional bias and emotional symptoms was sometimes revealed, which might be explained by the level of positive attentional biases in the participants [21, 31]. Therefore, involving only negative or positive attentional bias alone in the cognitive model of emotional symptoms might be problematic. Future endeavors might be put into examining the profiles of attentional biases in research to establish a cognitive model that depicted how the overall patterns of negative and positive attentional biases were related to emotional symptoms.

Moreover, results implied that clinicians should consider the overall patterns of negative and positive attentional biases when screening at-risk children during the COVID-19 pandemic. Clinicians might use the APNIS scale to facilitate the identification of children at higher risk of emotional symptoms during the COVID-19 pandemic. Children who simultaneously had patterns of high negative attentional bias and moderate positive attentional bias might be at higher risk of developing higher fear of COVID-19, anxiety symptoms, and depression symptoms. Thus, these children might need early prevention against more severe emotional problems in the future, addressing the maladaptive profiles of attentional biases.

### Limitations

The results should be interpreted with caution. The study only involved a group of Chinese children transiting into early adolescence. Thus, the results might not be generalized to other populations, such as late adolescents or adults. Moreover, a cultural factor might be a significant factor affecting the overall patterns of attentional biases. Thus, future studies might need to examine how the profiles of attentional biases are manifested in children from other cultures. Also, fear of COVID-19, anxiety symptoms and depression symptoms were measured by self-report inventories, which might be affected by children’s memory biases. Future studies might need to include multiple measures of anxiety symptoms and depression symptoms including physiological measures of anxiety symptoms and depression symptoms. The self-report assessment of attentional biases might only reflect children’s dispositional tendency to attend to negative or positive information. It should be noted that the cognitive resources allocation process underlying attentional biases might be better measured by the behavioral tasks. Thus, future studies might use the behavioral measures of attentional biases to examine the profiles of attentional biases in children. The longitudinal study only lasted for 6 months. The failure to find significant time effects and interaction effects on anxiety symptoms and depression symptoms might be due to the relatively short interval between the two assessments. Future studies might expand the assessment interval and/or involve more assessments in a longer period.

## Conclusions

Overall, the study identified three profiles of attentional biases: the “low negative and positive attentional biases” profile, the “moderate positive and high negative attentional biases” profile, and the “high positive and moderate negative attentional biases” profile. Children with the “moderate positive and high negative attentional biases” profile had significantly higher emotional symptoms during the COVID-19 pandemic. Psychologists may need to identify children with maladaptive profiles of attentional biases for early intervention. The results highlighted the importance of examining the overall patterns of negative and positive attentional biases when identifying at-risk children during the COVID-19 pandemic.

## Supplementary Information

**Additional file 1: Table S1.** Fit statistics for latent profile analysis models representing one to five class model. **Table S2.** Factor Loadings for Exploratory Factor Analysis With Varimax Rotation of the APNIS for the Chinese Children Sample (n=111).

## Data Availability

The datasets generated during and/or analyzed during the current study are available from the corresponding author upon reasonable request.
