# Supplementary material for: Patterns of attentional biases in children and emotional symptoms during the COVID-19 pandemic: a two-wave longitudinal study
Source: Child Adolesc Psychiatry Ment Health. 2023 May 17;17:61. doi: 10.1186/s13034-023-00594-y (PMC10189681; doi:10.1186/s13034-023-00594-y)
Supplement: Supplementary file 1 — Additional file 1: Table S1. Fit statistics for latent profile analysis models representing one to five class model. Table S2. Factor Loadings for Exploratory Factor Analysis With Varimax Rotation of the APNIS for the Chinese Children Sample (n=111). [file 13034_2023_594_MOESM1_ESM.docx]

**Additional file Information**

**S.Table 1. Fit statistics for latent profile analysis models representing one to five class model**

| Number of groups | BIC | AIC | VLMR  (p-Value) | Adj. LMR (p-Value) | BLRT  (p-Value) | Entropy |
| --- | --- | --- | --- | --- | --- | --- |
| 1 | 2863.28 | 2848.98 | NA | NA | NA | NA |
| 2 | 2868.41 | 2843.38 | .09 | .10 | 0.00 | 0.95 |
| 3 | **2861.14** | **2825.39** | **0.03** | **0.04** | **0.00** | **0.77** |
| 4 | 2872.96 | 2826.47 | 0.35 | 0.37 | 0.50 | 0.71 |

*Note.* The bolded four-group model showed the best model fit.

**Exploratory Factor Analysis**

Exploratory factor analysis (EFA) was conducted to explore the factor structure of the APNIS with SPSS version 22 with an independent sample of 120 children (age from 11 to 13 years). First, the Kaiser–Meyer–Olkin (KMO) test was performed. KMO value was .773, and Bartlett’s test of Sphericity was significant, *χ2*= 2187.350, *df*=780, *p*< .001, suggesting good factorability. This suggested that Principle Component Analysis (PCA) is suitable to explore the factor structure of the scale. Thus, PCA with Varimax rotation was conducted. The first PCA revealed 10 factors, which accumulatively accounted for 66.54% of the variance. 20 items had cross-loadings with a difference below .30, which were deleted. The second PCA displayed that 4 items had cross-loadings, which were also deleted. The third PCA showed that another four items had cross-loadings. These items were subsequently deleted. The fourth PCA showed that four items were loaded on factors that had fewer than three items. Thus, these factors were deleted. The final PCA showed a clear two-factor structure that had eigenvalues above 1. The first factor was defined as “positive attentional bias”. It included items 13, 17, 18, and 40, accounting for 28.226% of the variance. The second factor was named “negative attentional bias” which included item 2, item 4, item 5, and item 22. This factor explained 36.501% of the variance. The two-factor model explained 64.726% of the variance.

**Confirmatory Factor Analysis**

We conducted CFA to confirm the two-factor structure of APNIS with the sample of 264 children using AMOS 24 (Arbuckle, 2014). The fit indices revealed that the structural validity of the two-factor 8-item brief APNIS for children was good (RMSEA =.065; GFI=.963, AGFI=.934; CFI=.935, TLI=.909) (Bentler & Bonett, 1980; Browne & Cudeck, 1989; Kline, 2005). Although the Chi-Square Test of Model Fit was significant, *χ^2^* (20) = 42.545, *p*<.005, this index tends to be significant in a large sample (Schumacker & Lomax, 2010).

**S.Table 2.** Factor Loadings for Exploratory Factor Analysis With Varimax Rotation of the APNIS for the Chinese Children Sample (n=111).

| Items | Factor 1 | Factor 2 |
| --- | --- | --- |
| Factor 1: Negative attentional bias |  |  |
| Item 2 I can’t forget the times I have performed poorly at something. 我无法忘记自己在某些事情上表现不好的时候。 | .823 |  |
| Item 4 I am particularly aware of the bad news that  appears in TV news broadcasts我会特别注意电视新闻上  的坏消息 | .787 |  |
| Item 5 Things that I am not good at are always on my mind那些我做得不好的事情总是萦绕在我的脑海中。 | .873 |  |
| Item 22 I usually notice situations that made me feel bad in the past我经常注意过去那些令自己不快乐的情境。 | .826 |  |
| Factor 2: Positive attentional bias |  |  |
| Item 13 I pay attention to my positive characteristics of myself. 我会注意到自己的正面特质。  Item 17 I realize and pay attention to moments when everything is going well. 我会察觉并注意到事事顺利的时刻。 |  | .814  .879 |
| Item 18 I am particularly aware of things that I am  successful at. 我特别留意自己做得成功的事情。  Item 40 It is important for me to remember the good  things about others. 对我来说, 认为别人好很重要。 |  | .873  .381 |
|  |  |  |
